# Quantitative myocardial perfusion in mice based on the signal intensity of flow sensitized CMR

**DOI:** 10.1186/1532-429X-14-73

**Published:** 2012-10-24

**Authors:** Sumeda Abeykoon, Michelle Sargent, Janaka P Wansapura

**Affiliations:** 1 Department of Physics, University of Cincinnati, Cincinnati, OH, USA; 2 Imaging Research Center, Department of Radiology, Cincinnati Children's Hospital, 3333 Burnet Ave, MLC 5033, Cincinnati, OH, 45229, USA; 3 Howard Hughes Medical Institute, Molecular Cardiovascular Biology, Cincinnati Children Hospital, Cincinnati, OH, USA

**Keywords:** Myocardial perfusion, T1, Mice, Arterial spin labeling

## Abstract

**Background:**

In the conventional approach to arterial spin labeling in the rodent heart, the relative difference in the apparent *T*_*1*_ relaxation times corresponding to selective and non-selective inversion is related to perfusion via a two compartment model of tissue. But accurate determination of *T*_*1*_ in small animal hearts is difficult and prone to errors due to long scan times and high heart rates. In this study we introduce the theoretical frame work for an alternative method (*SI-method*) based purely on the signal intensity of slice-select and non-select inversion recovery images at a single inversion time at short repetition time.

**Methods:**

A modified Bloch equation was solved to derive perfusion as a function of signal intensity of flow sensitized segmented gradient echo acquisitions. A two compartment fast exchanging model of tissue was assumed. To test the new technique first it was implemented on a flow phantom and then it was compared with the conventional *T*_*1*_ method in an *in vivo* study of healthy C57BL/6 mice (n=12). Finally the *SI-method* was used in comparison to a Late Gadolinium Enhanced (LGE) method to qualitatively and quantitatively assess perfusion deficits in an ischemia-reperfusion mouse model (n=4).

**Results:**

The myocardial perfusion of healthy mice obtained by the *SI-method*, 5.6 ± 0.5 ml/g/min, (mean ± standard deviation) was similar (p=0.38) to that obtained by the conventional method, 5.6 ± 0.3 ml/g/min. The variance in perfusion within the left ventricle was less for the *SI-method* than that for the conventional method (p<0.0001). The mean percentage standard deviation among repeated measures was 3.6%. The LGE regions of the ischemia reperfusion model were matched with regions of hypo-perfusion in the perfusion map. The average perfusion in the hypo perfused region among all four IR mice was 1.2 ± 0.9 ml/g/min and that of the remote region was 4.4 ± 1.2 ml/g/min.

**Conclusions:**

The proposed signal intensity based ASL method with a segmented acquisition scheme allows accurate high resolution perfusion mapping in small animals. It’s short scan time, high reproducibility and ease of post process makes it a robust alternative to the conventional ASL technique that relies on T_1_ measurements.

## Background

To quantify perfusion, the Arterial Spin Labeling (ASL) technique uses water protons as a freely diffusible contrast agent. The contrast due to perfusion is obtained by preparation of magnetization by selective and non-selective inversion. Slice-selective inversion causes signal enhancement due to in-flow of thermally stabilized spins whereas global inversion is insensitive to flow. In the cardiac implementation of ASL, spin labeling is generally done within the detection slice. One approach of ASL is to mathematically relate the relative difference in the apparent relaxation times corresponding to selective and non-selective inversion, to perfusion via a two compartment model of tissue [1]. In this case perfusion, *P*, is given by:

(1) $$P=\frac{\lambda }{T_{1c}}\left(\frac{T_{1g}}{T_{1s}}-1\right)$$

Where, T_ls_ and T_lg_ are the apparent longitudinal relaxation times for slice select and non-select inversion respectively. T_lc_ is the longitudinal relaxation time of blood. This ASL method, hereafter referred to as the *T1-method*, has been used to quantify myocardial perfusion in humans [2,3] as well as in small animal models [4-7]. Accurate determination of T_l_ relaxation times require long scan time to allow for full relaxation of the longitudinal magnetization. Long scan times in turn leads to varied inversion times for different read outs of the k-space in segmented acquisitions in the heart, causing errors in T_l_ maps and therefore in perfusion maps.

In this study we propose an alternative ASL method, which we will call the *SI-method*, based purely on the signal intensity of slice-select and non-select inversion recovery images at a single inversion time at short repetition time. A steady state image is also acquired to normalize the receiver characteristics. A new expression for perfusion is derived for this acquisition scheme assuming a two compartment model of tissue [1]. Similar signal intensity based methods with single inversion times have been used in the past in brain and cardiac perfusion imaging [8-11]. A theoretical frame work for ASL with short repetition time has also been introduced by Pell et al. [11]. What is different in the current study is the use of short repetition time approach combined with a dedicated two-compartment theory.

We adapted the two compartment model proposed by Bauer et al. [1] and derived perfusion as a function of signal intensity of slice select and non-select inversion acquisitions by solving a modified Bloch equation. To test the new technique we first implemented it on a flow phantom with two compartments with no exchange between them. Since it is difficult to accurately quantify spin exchange rates in materials, this phantom experiment allowed us to know perfusion exactly given a specific rate of flow. Second, we compared the proposed *SI-method* with the conventional *T1-method* by carrying out *in vivo* myocardial perfusion measurements in healthy mice. Finally the *SI-method* was used in comparison to a LGE method to qualitatively and quantitatively assess perfusion deficits in an ischemia-reperfusion mouse model.

## Methods

### *SI-method* for a fast exchanging two compartment model of tissue

**Figure 1:**
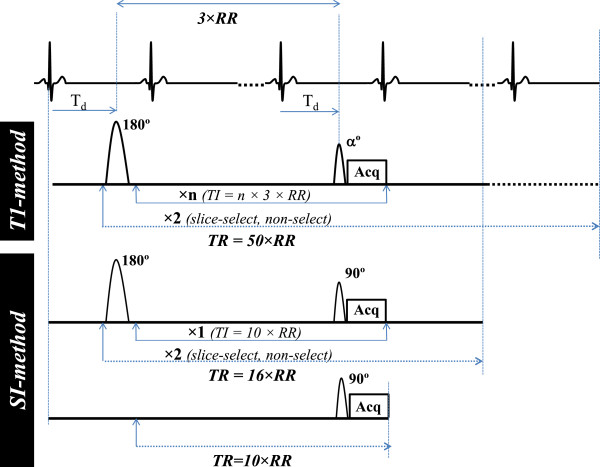
**Acquisition schemes for the mouse cardiac studies.** Slice select and non-select, segmented, Look-Locker inversion recovery sequences with data acquisitions at every third cardiac cycle was used for the conventional *T1-method* (top). The TR was set to 50 RR intervals in this case. The *SI-method* (bottom) consisted of three acquisitions: Slice select and non-select, segmented, inversion recovery sequences, each with one acquisition at 10×RR and a segmented gradient echo sequence with no spin preparation and TR set to 10×RR. In both methods the acquisition block comprised a single excitation pulse followed by one line of k space.

**Figure 2:**
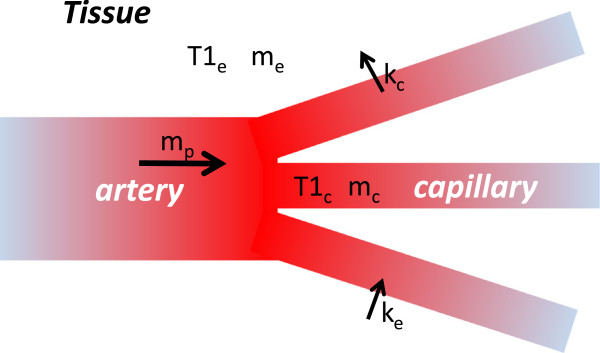
**The two compartment model.** Myocardial tissue consists of homogenous intra and extra-capillary space with respect to relaxation time. *T*_1*e,c*_, *m*_*e,c*_ and *k*_*e,c*_ are longitudinal relaxation time, magnetization, and spin exchange rate in the intra-capillary region (susbscript c) and extra-capillary region (subscript e). *m*_*p*_(*t*) is the magnetization of the incoming blood.

The data acquisition scheme for this method consists of three scans: (i) Slice selective inversion-TI-acquisition, (ii) Non-select inversion-TI-acquisition, (iii) steady state acquisition with TR set to TI. The method as it was implemented in mice is depicted in Figure 1. A segmented k-space acquisition is assumed. A finite TR is assumed for (i) and (ii). We will derive an expression for magnetization for each of these cases in terms of perfusion in a two compartment model of tissue (Figure 2) where we assume myocardial tissue to be consisting of homogenous intra and extra-capillary space with respect to relaxation time. The rate of change of magnetization in the intra-capillary region m_c_(t) depends on loss of magnetization due to T_1_ relaxation, exchange of magnetic spins between intra and extra-capillary region and gain of magnetization due to in flow of blood. The magnetization in the extra-capillary region m_e_(t) is unaffected by blood flow. The spin dynamics of the two compartments is described by the following modified Bloch equations.

(2) $$\begin{array}{c} \frac{dm_{c}\left(t\right)}{dt}=\left(\frac{m_{c}\left(0\right)-m_{c}\left(t\right)}{T_{1c}}\right)+\frac{Fm_{p}}{V_{c}}-\frac{Fm_{c}\left(t\right)}{V_{c}}\\ \;-K_{c}m_{c}\left(t\right)+K_{e}m_{e}\left(t\right) \end{array}$$

(3) $$\frac{dm_{e}\left(t\right)}{dt}=\left(\frac{m_{e}\left(0\right)-m_{e}\left(t\right)}{T_{1e}}\right)+K_{e}m_{c}\left(t\right)-K_{e}m_{e}\left(t\right)$$

Where T_lx_, m_x_(t), V_x_ and K_x_ are longitudinal relaxation time, magnetization, volume, spin exchange rate respectively with the subscript *x=c* for intra-capillary region and *x=e* for extra-capillary region. m_p_(t) is the magnetization of the incoming blood. The total magnetization of tissue, M(t), is calculated as,

(4) $$M\left(t\right)=\left(\frac{RBV}{\lambda }\right)m_{c}\left(t\right)+\left(1-\frac{RBV}{\lambda }\right)m_{e}\left(t\right)$$

where *λ* is the blood tissue water partition coefficient [4], $RBV=\frac{V_{c}}{V_{t}}$ and *V*_*T*_ = *V*_*c*_ + *V*_*e*_. Equations 13 describe the time evaluation of the total magnetization of tissue and can be solved for a given input magnetization, m_p_(t), depending on the type of spin preparation. For slice-select inversion, m_p_(t) is the equilibrium magnetization of capillary blood hence is equal to the constant m_c_(0) in magnitude. For non-select inversion, at steady state, $m_{p}\left(t\right)=m_{c}\left(0\right)\left(1-2e^{-\frac{TI}{T_{1c}}}\right)+m_{c}\left(0\right)e^{-\frac{TR}{T_{1c}}}$, where *TI* and *TR* are the inversion and repetition times respectively (see Appendix).

The total magnetization for each spin preparation can be written in matrix from as follows:

(5) $$\frac{d}{dt}\left(M_{s}\left(t\right)e^{-Rt}\right)=\frac{P}{RBV}e^{-Rt}m_{c}\left(0\right)+e^{-Rt}\left(\begin{array}{c} \frac{m_{c}\left(0\right)}{T_{1c}}\\ \frac{m_{e}\left(0\right)}{T_{1e}} \end{array}\right)$$

for slice-select inversion and

(6) $$\begin{array}{c} \frac{d}{dt}\left(M_{g}\left(t\right)e^{-Rt}\right)=\frac{P}{RBV}m_{c}\left(0\right)\left(1-2e^{-\left(\frac{TI}{T_{1c}}\right)}+e^{-\left(\frac{TR}{T_{1c}}\right)}\right)e^{-Rt}\\ \;+e^{-Rt}\left(\begin{array}{c} \frac{m_{c}\left(0\right)}{T_{1c}}\\ \frac{m_{e}\left(0\right)}{T_{1e}} \end{array}\right) \end{array}$$

for non-select inversion, where

(none1) $$R=\left[\overset{-\left(\frac{1}{T_{1c}}+\frac{F}{V_{c}}+K_{c}\right)}{K_{e}}\overset{K_{c}}{-\left(\frac{1}{T_{1e}}+K_{e}\right)}\right]$$

Equations (5) and (6) can be solved to derive the magnetization for slice-select M_s_(t=TI) and non-select inversion M_g_(t=TI) using the initial condition that all magnetization within the selected slice is inverted at t=0 in both preparation schemes. The term *R* can be simplified following a series of steps proposed by Bauer et al. [1,12] assuming fast exchange between intra and extra capillary regions i.e. Kc≈Ke≈∞. This assumption reduces the complexity of the model but is appropriate for myocardial tissue that has a high exchange rate compared to other tissues of the body [12]. The difference in the signal intensities between the two preparation schemes can now be defined as *ΔM* = *M*_*g*_(*t* = *TI*) − *M*_*s*_(*t* = *TI*). By combining the simplified expression for *R* and the solutions to equations (5) and (6) we obtain,

(7) $$\Delta M=\frac{2Pm_{c}\left(0\right)\left(e^{-\left(\frac{TI}{T_{1c}}\right)-e^{-\left(\mu ,T,I\right)}}\right)}{\left(\mu -\frac{1}{T_{1c}}\right)\lambda }+\frac{Pm_{c}(0)e^{-(\frac{TR}{T_{1c}})}(e^{-\mu TI}-1)}{\mu \lambda }$$

Where, $\mu =\left(\frac{RBV}{\lambda }\right)\frac{1}{T_{1c}}+\left(1-\frac{RBV}{\lambda }\right)\frac{1}{T_{1e}}+\frac{P}{\lambda }$

To remove the dependency on receiver characteristics, we normalize ΔM by the magnetization of a steady state gradient echo acquisition (M_s_) with no spin preparation and with *TR* set to the same *TI* as in equation (7) (Figure 1). Since echo time of this acquisition is small (~ 1ms) we assume that *M*_*s*_ is not affected by perfusion. We also assume equilibrium magnetization of intra and extra-capillary regions to be similar i.e. *m*_*c*_(0) ≈ *m*_*e*_(0). Thus we obtain,

(8) $$M_{s}=m_{c}\left(0\right)\left(1-e^{-\mu_{0}TI}\right)$$

Where, $\mu_{0}=\left(\frac{RBV}{\lambda }\right)\frac{1}{T_{1c}}+\left(1-\frac{RBV}{\lambda }\right)\frac{1}{T_{1e}}=\frac{1}{T_{1}}$

Here *T*_*1*_ is the myocardial longitudinal relaxation time without the in-flow effects. By dividing equation (7) by (8) we obtain an expression for the normalized magnetization difference, ΔM/M_s_ Then by taking the first order Taylor expansion of ΔM/M_s_ about *TI* we obtain,

(9) $$P=\frac{\frac{\lambda \Delta M}{M_{s}}}{\left(1-e^{-\frac{TR}{T_{1c}}}+e^{-\frac{TI}{T_{1c}}}\right)T_{1}}$$

### *SI-method* for a non-exchangeable two compartment model of tissue

Perfusion is derived for a non-exchangeable model of two compartments based on the same acquisition method as described above. The aim is to test the *SI-method* on a flow phantom. Since water exchange rates of tubing material is difficult to assess we chose material with zero permeability. This allows perfusion to be known exactly in terms of volume of fluid / volume of phantom / minute (i.e. min^-1^), given a specific flow rate. The phantom will have two compartments, intra- and extra capillary, but with no exchange between them. Accordingly the *SI-method* is modified for this scenario by making K_c_=0, K_e_=0 in equations (2) and (3). The rest of the derivation is similar to as in the previous section.

The difference in the signal intensities between the two preparation schemes, ΔM, is obtained by first order Taylor expansion as in the previous section. By solving for perfusion we get,

(10) $$P=\frac{\lambda \Delta M}{m_{c}\left(0\right)\left(2e^{-\frac{TI}{T_{1c}}}-e^{-\frac{TR}{T_{1c}}}\right)TI}$$

Here we do not normalize *ΔM* by the signal intensity of a steady state acquisition, instead measure m_c_(0)*,* the equilibrium magnetization of the capillary fluid*,* using a spin echo sequence with long *TR*.

### *T1-method* for a non-exchangeable two compartment model of tissue

The aim is to compare the results of the *T1-method* with that of the *SI-method* using the flow phantom described above. The *T1-method* was originally developed by Bauer et al. [4] for a fast exchanging two compartment model of tissue. Here we modify it to reflect no exchange between compartments. Again we begin with setting K_c_=0, K_e_=0 in equations (2) and (3). In the *T1-method* we are interested in the apparent longitudinal relaxation times and not the magnitudes of magnetization. To make the derivation of relaxation times easier we normalize the magnetization of intra and extra-capillary regions at the time of inversion to one. The equilibrium magnetization on the other hand is set to zero i.e. m_c_(0)=m_e_(0)=0 in equations (2) and (3). The total magnetization of tissue, M(t), is given by equation (4) as before. Modified Equations 24 can be solved for a given input magnetization, m_p_(t), depending on the type of spin preparation. According to the normalizing scheme introduced here the input magnetization, m_p_(t)*,* for slice-select inversion is zero and $m_{p}\left(t\right)=e^{-\frac{t}{T_{1c}}}$, for non-select inversion.

By taking into account the mean relaxation time approximation that *T*_1_ = ∫ _0_^*∞*^M(*t*)*dt*, [4,13], we obtain the following expressions for apparent relaxation times

(11) $$T_{1s}=\frac{RBV}{\lambda }\left(\frac{RBVT_{1c}}{PT_{1c}+RBV}\right)+\left(1-\frac{RBV}{\lambda }\right)T_{1e}$$

for slice-select inversion, and

(12) $$T_{1g}=\frac{RBV}{\lambda }T_{1c}+\left(1-\frac{RBV}{\lambda }\right)T_{1e}$$

for non-select inversion. From equations (11) and (12) we derive an expression for perfusion in a two compartment model of tissue with no exchange.

(13) $$P=\frac{1}{T_{1c}\left\{\frac{1}{\lambda }\left(\frac{T_{1c}}{T_{1g}-T_{1s}}\right)-\frac{1}{RBV}\right\}}$$

The *RBV* in the context of the said phantom is equal to the volume ratio V_c_/V_T_, where *V*_*c*_ is the volume of capillary tube and *V*_*T*_ is the total volume of the phantom as measured from a cross section of the phantom.

### Two compartmented flow phantom study

A phantom was prepared by placing 12 loops of capillary tube (Cole Parmer # EW 06492–02) with inner diameter 0.51 mm, inside a 3cc syringe. One end of the capillary tube was fitted to a syringe infusion pump (Model R-99E, Razel Scientific Instruments, St. Albans, VT). The space between capillary tubes was filed with a mixture of Gadolinium and Poly Vinyl Alcohol solution. Diluted Gd solution was used as the capillary liquid. Intra and extra-capillary liquids were prepared to have approximately similar longitudinal relaxation times of blood and tissue at 7 Tesla (*T*_*1-intra*_ = 1896 ms, *T*_*1-extra*_ = 1344 ms). Perfusion was calculated using equation (10) for the *SI-method* and equation (13) for the *T1-methods* for the following flow velocities 0.03, 0.07, 0.09, 0.1, 0.18, 0.3, 0.35 cm s^-1^. The average signal intensity of a region covering the phantom was considered for calculation of *ΔM* and for curve fitting of *T*_*1*_.

Cardiovascular magnetic resonance (CMR) was performed on a 7T Bruker Biospec system using an inversion recovery FLASH sequence consisting of a sinc3 excitation pulse and a hyperbolic secant pulse (sech) for inversion. For the *T1-method*, slice select inversion and non-select inversion prepared images were acquired at a constant repetition time, varying the inversion time, *TI*, in twelve steps between 50 and 5500 ms. *TR/TE* = 6000 ms/ 2.6 ms field of view = 2.5 × 2.5 cm^2^, flip angle = 30°, slice thickness = 4 mm, a matrix size = 256 × 64. For the *SI-method*, TR = 1000 ms and *TI* = 100 ms, flip angle = 90°, All other imaging parameters were set similar to that of the *T1-method*. Imaging was performed perpendicular to the direction of flow. For both methods, the average signal intensity of a region of interest covering a cross section of the phantom was considered for calculations. *T*_*1s*_ and *T*_*1g*_ were calculated by fitting to a mono exponential function of the form $Ae^{-\frac{TI}{T_{1}}}+B$. Spin density of intra (ρ_i_) and extra-capillary (ρ_e_) liquids were obtained by a spin echo pulse sequence with *TR*=20 sec and with all other imaging parameters including receiver gains fixed. Perfusion was calculated using equation (10) for *SI-method* and equation (13) for *T1-method* taking *λ* = (*ρ*_*i*_ + *ρ*_*e*_)/*ρ*_*i*_ and *m*_*c*_(0) = *ρ*_*i*_.

### *In vivo* mouse study

The study was conducted under a protocol approved by the Institutional Animal Care and Use Committee. To compare the proposed *SI-method* with the conventional *T1-method*, we measured myocardial perfusion of healthy ten weeks old C57BL/6 mice (n=12) using both methods. Mice were anesthetized with continuous inhaled Isoflurane (2% by volume) administered via a nose cone. Constant body temperature of 37°C was maintained using a thermocouple/heater system. Both SI and T1 methods were performed in the same session. To test the reproducibility of the *SI-method* three perfusion measurements were made on the same mouse. Each measurement was performed on a separate session (often on a different day). Three C57BL/6 mice were used for this study. Finally we used the *SI-method* to quantify perfusion in a mouse model of ischemia-reperfusion (IR). Four C57BL/6 mice were anesthetized with isoflurane and intubated through the mouth and ventilated. The left coronary artery was ligated with a silk ligature following thoracotomy. The animals were maintained in the ligated state for 30 minutes, after which the externalized silk was pulled to release the constriction and hence allowing full reperfusion. IR mice were scanned within 24 hours of surgery. The *in vivo* study was performed on a 7T Bruker Biospec system using a custom made solenoid coil having a single turn consisting of a wide conductive sheet (diameter =3.0 cm, length =3.5 cm). The sensitivity of the coil covered approximately 80%- 90% of the mouse’s body. In IR mice, in addition to myocardial perfusion measurement, LGE experiment was performed 30 minutes after injecting a bolus of Gd-DTPA (0.3-0.6 mmol/kg) intraperitonealy. *T1* weighted cine imaging was performed in the short axis using a segmented FLASH sequence to highlight enhancement. Slice thickness=1.0 mm, matrix size=256x256, in-plane resolution=117x117 μm2. TE/TR=3/5.2ms, flip angle=30º. Image acquisition was prospectively ECG gated using pediatric ECG probes attached to the paws. A pneumatic pillow was used for respiratory gating (SA Instruments, Stony Brooks, NY).

The acquisition schemes for *T1* and *SI methods* are shown in Figure 1. A gated, Look-Locker sequence [5] was implemented for the *T1-method*. A 5ms hyperbolic secant pulse was used for inversion. For the *T1-method*, for each inversion, the relaxation curve was sampled at twelve different inversion times at every third cardiac cycle i.e. *TI* = 3 × *n* × *RR*, *n* = 1,.12 with *R* ≈ 50 × *RR*, that is approximately 6 seconds. For the *SI-method* the same sequence was used with data acquired at a single *TI* time of 10×RR with TR=16×RR, that is approximately 2 seconds. In both methods the acquisition block comprised a single excitation pulse followed by one line of k space. For the *SI-method* an additional steady state gradient echo image was acquired without the inversion pulse with TR=10×RR. All images were acquired in the short axis plane at end diastole. The following common imaging parameters were used: TE = 1.73 ms, field of view = 2.5 × 2.5 cm^2^, slice thickness = 2.0 mm, matrix size = 128 × 64, flip angle = 90° and 10° were used for SI and T1 method respectively. For slice select inversion, the slice thickness of the inversion pulse was set to twice that of the excitation slice to eliminate effects from imperfect edges of both the inversion and the imaging pulse profiles

For the *in vivo* study we assumed myocardial tissue to consist of fast exchanging two compartments. Therefore we used equation (9) for the *SI-method*, and equation (1) for the *T1-method* for calculating perfusion. Following constant values were used: T_1_ of blood, T_1c_ = 1800 ms, T_1_ of myocardial tissue in the absence of flow effects, T_1_ = 1400 ms, λ=0.95 ml/g [14]. Perfusion was calculated on a pixel-by-pixel basis. The mean value was calculated from an ROI drawn within the left ventricle wall. Pixel values less than −5 ml/g/min and greater than 15 ml/g/min were set to zero and also threshold limit was set to obtain a noise free perfusion map. The apparent *T*_*1*_ values obtained by the Look Locker acquisition was corrected for saturation effects as previously described [5].

Results are expressed as mean ± standard deviation. Statistical differences were assessed with the unpaired 2-tailed Student’s t test for two experimental groups. A nonparametric test was applied when the data were not normally distributed. A 2-tailed P value of less than 0.05 was considered statistically significant.

## Results

**Figure 3:**
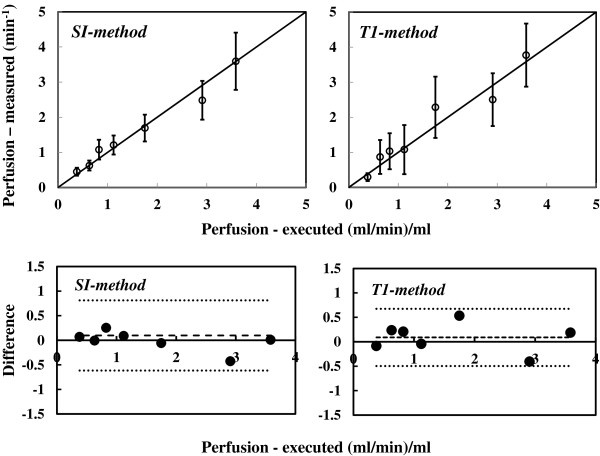
**Comparison of phantom results.** The perfusion measured by *SI-* (left) and *T1-methods* (right) is compared with that calculated from the flow rates. The Bland Altman plots show that the difference between the executed perfusion and the measured are within the 95% confidence level in both cases.

The results of the phantom experiments are shown in Figure 3. In this case the perfusion is expressed as the flow rate per unit volume of phantom i.e. in (ml/min)/ (ml). Perfusion measured by both methods agrees well with that calculated using the flow rates in the capillary tubes. The deviation from the actual perfusion is greater for higher flow rates for both methods, the maximum being 16%. The Bland Altman plots show that the difference between the executed perfusion and the measured stay within the 95% confidence level in both cases.

The error bars shown in Figure 3 are derived by propagating errors of *P* in equations (10) and (13) for *SI* and *T1 methods*. Larger errors in the T1-method reflect greater uncertainty of *T*_*1s*_ and *T*_*1g*_ measurements.

**Figure 4:**
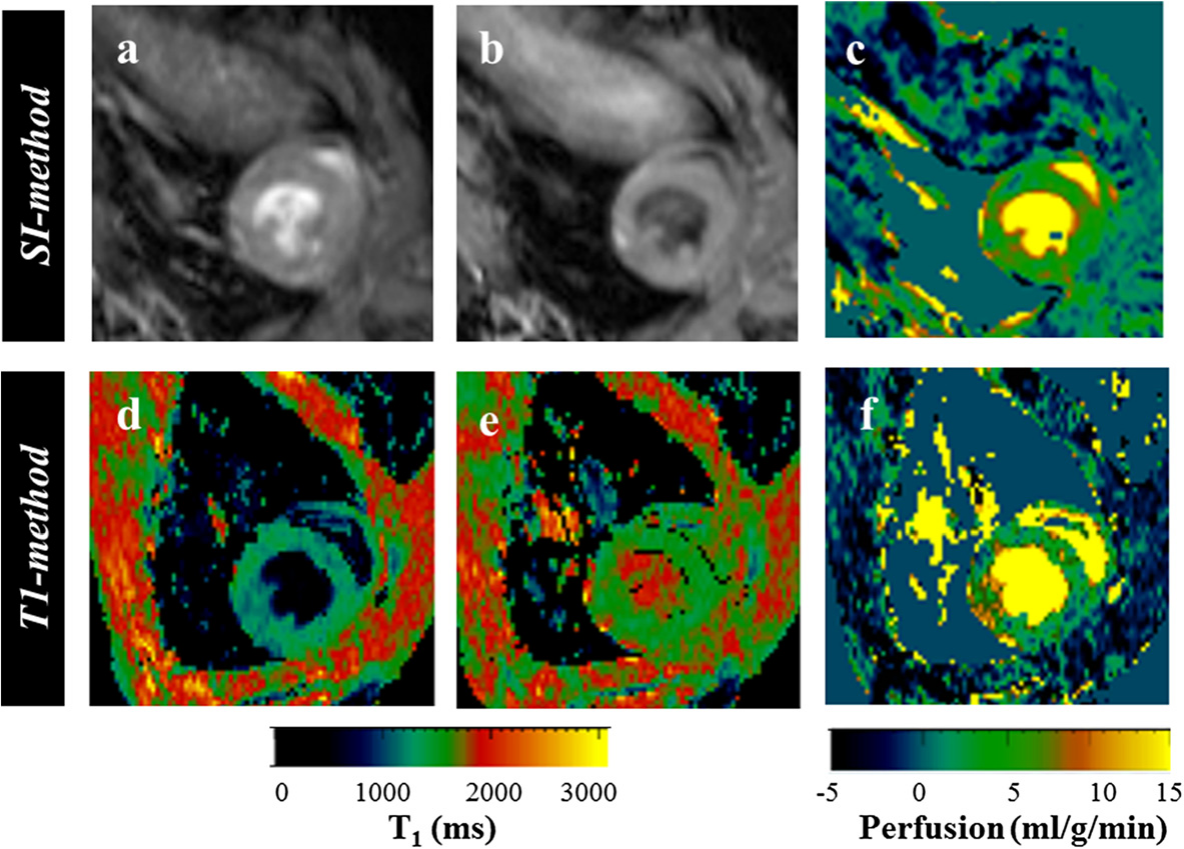
**Comparison of*in vivo*perfusion maps.** Example of raw images and calculated perfusion maps of the left ventricle in short axis plane are shown for each technique. *SI-method*: (**a**) slice-select inversion image, (**b**) non-select inversion image, (**c**) perfusion. *T1-method*: (**d**) T_1s_ map, (**e**) T_1g_ map, (**f**) perfusion.

Typical left ventricular perfusion maps of a normal mouse, by *SI* and *T1-method* are given in Figure 4. The average *T*_*1s*_ and *T*_*1g*_ for normal mice in our study was 1293 ± 80 ms and 1542 ± 88 ms respectively. The Table 1 compares the perfusion values obtained by the two methods for each mouse. Perfusion was calculated from region of interest covering the entire left ventricle wall. The myocardial perfusion of healthy mice obtained by the *SI-method* is 5.6 ± 0.5 ml/g/min, (mean ± standard deviation). This is not significantly different (p>0.05) to that obtained by the T1 method, 5.6 ± 0.3 ml/g/min. The standard deviation given for each mouse in Table 1 reflects the variation of *P* within the left ventricle for each mouse. The mean value of this standard deviation is significantly higher for the *T1-method* (2.3 ml/g/min) compared to that of the *SI-method* (1.6 ml/g/min), p<0.0001. As seen in Table 2 the mean percentage standard deviation among repeated measures is less than 5% for the *SI-method*. 

**Table 1:** **Comparison of*in vivo*results for each mouse**

**Mouse ID**	***SI-method*(ml/g/min)**		***T1-method*(ml/g/min)**	
	**Mean**	**Standard deviation**	**Mean**	**Standard deviation**
1	5.8	2.0	5.6	2.9
2	5.6	1.8	5.7	2.7
3	5.7	1.8	5.4	2.1
4	6.4	1.9	6.1	2.2
5	6.2	1.8	5.5	2.9
6	4.8	1.3	5.1	2.1
7	5.5	1.8	5.6	2.0
8	5.9	1.6	6.1	2.2
9	5.7	1.8	5.9	2.5
10	5.3	1.3	5.3	2.1
11	5.8	1.7	5.6	2.5
12	4.8	1.3	5.0	2.2
Average	** 5.6**±**0.5**	**1.6**	** 5.6±0.3**	**2.3**

**Table 2:** **Reproducibility of the*SI-method***

**Mouse ID**	**Perfusion (ml/g/min)**				(std. dev/mean)*100
	**exp. 1**	**exp. 2**	**exp. 3**	**Mean ± std. dev**	
1	4.8	5.0	5.2	5.0 ±0.2	4%
2	5.3	5.8	5.7	5.6 ±0.2	4.8%
3	6.2	6.3	6.4	6.3 ±0.1	2%

**Figure 5:**
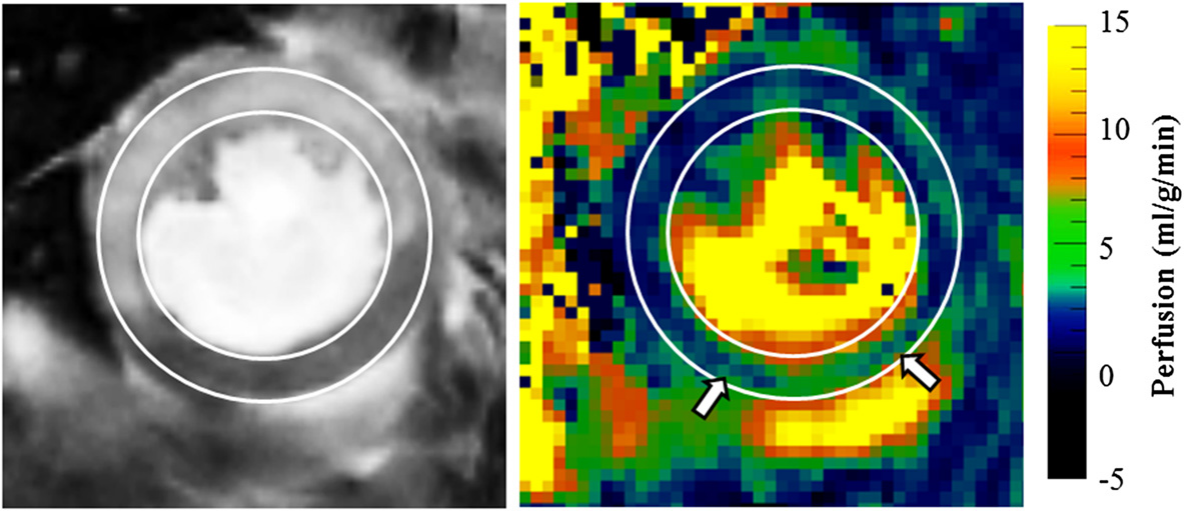
**Perfusion in the ischemia-reperfusion model.** LGE in the lateral wall and the anterior septum (left) are matched with regions of hypo-perfusion in the perfusion map (right). Arrows show low perfusion regions extending to the mid-myocardium beyond the boundary of the enhancement.

Figure 5 shows an example of LGE and perfusion in an IR mouse in the short axis plane. The enhancement in the lateral wall and the anterior septum are matched with regions of hypo-perfusion in the perfusion map. The average perfusion in the hypoperfused region among all four IR mice was 1.2 ± 0.9 ml/g/min and that of the remote region was 4.4 ± 1.2 ml/g/min.

## Discussion

In this study, a new ASL method to quantify myocardial perfusion is introduced based on the signal intensity of flow sensitized CMR. Following Bauer’s [1] frame work, myocardium is modeled as a two compartment with respect to *T*_*1*_, which takes in to consideration that *T*_*1*_ of capillary blood is different to that of tissue. The imaging method consists of segmented, single gradient echo acquisitions prepared by slice select and non-select inversion pulses with finite repetition times followed by a gradient echo acquisition with no spin preparation. A simple expression for perfusion was derived by solving a modified Bloch equation for this model.

An important feature of our tissue model is that it consists of two compartments. In contrast, the Detre’s frame work [15] and its derivative ASL methods by Kwong [9], Buxton [8] and Zun [10] considers the tissue to be consists of a single compartment of blood. However, because *T*_*1*_ of blood is higher than that of tissue, taking tissue to be a single compartment of blood could lead to overestimation of perfusion. This is problematic if absolute values of perfusion are desired. On the other hand, we can arrive at the same expression for *P* as did Kwong and others by reducing our model to a single compartment model. To achieve this we take the two compartment non-exchange version of the *SI-method* (Equation 10) which is similar to one compartment of blood as far as *ΔM* is concerned. Then, by making *TR* → *∞* in equation 10 we obtain the same expression for *P* as in equation 5 of reference 9. Because of the two compartment model, the *SI-method* requires the knowledge of *T*_*1*_ of tissue with no in-flow effects (*T*_*1*_) (Equation (9)) in addition to the *T*_*1*_ of blood (*T*_*1c*_) whereas Detre’s method only requires the knowledge of *T*_*1c*_. In this paper we have presented *P* calculated using literature values of *T*_*1*_ and *T*_*1c*_. However, in our case these relaxation times could also be calculated from data acquired for the *T1-method*. Hence we calculated *T*_*1*_ and *T*_*1c*_ for each mouse in Table 1 and used it to derive *P* using the *SI-method* (data not shown). The average *P* calculated using individually assessed *T*_*1*_ and *T*_*1c*_ was 5.7 ± 0.4 ml/g/min. Since this is not significantly different to the average *P* calculated using literature values (=5.6 ± 0.5 ml/g/min) it appears that the variability of perfusion among mice is greater than the uncertainty posed by *T*_*1*_ and *T*_*1c*_ in the *SI-method*.

The other important feature of the *SI-method* is that its k-space acquisition scheme is segmented as opposed to a single shot acquisition. Also, in deriving *P* no assumption was made about the length of the repetition time. This allows phase encoding steps to be acquired in multiple inversions with shorter TR times than it is required for full relaxation. Unlike in humans, the cardiac cycle in small animals is too short (~ 150 ms under anesthesia) to acquire a full set of k-space data in a single inversion. Therefore, by allowing for short repetition times, the *SI-method* considerably shortens the total acquisition time for high resolution experiments. Depending on the heart rate, the total scan time for the *T1-method* ranged from 6–8 minutes while that for the *SI-method* was 2–4 minutes. In human studies, the single shot acquisition limits the in-plane resolution since all k-space data must be acquired in a short period of time in diastole. In this context the segmented acquisition scheme of the *SI-method* and its theoretical model allows to achieve high resolution perfusion maps if the temporal noise could be mitigated.

The phantom and the *in vivo* study results show that the *P* values measured with the *SI-method* is comparable with that measured with the conventional *T1-method*. With the flow phantom we were able to verify the accuracy of the *SI-method* albeit with no spin exchange effects. In order to apply the *SI-method* to the flow phantom, the theoretical model was modified to reflect spin non-exchangeability. In this respect, the phantom is an exact physical replica of the modified theoretical model. Therefore the accuracy of the predicted outcome carried no uncertainty due to the design of the model. Hence the phantom experiment was an ideal method to test the relationship of *P* to flow sensitized MR signal as predicted by physics. However, in reality it is difficult to ascertain how well the two compartment model mimic myocardial tissue.

The fluorescent microspheres or fluospheres, have recently become a popular alternative to radioactive microspheres for quantitating myocardial perfusion in rodent experimental studies. The myocardial perfusion of healthy C57BL/6 mice measured using fluorescent microspheres (5.7 ± 0.3 ml/g/min**)** by Richer et al. [16] closely matches that of the *SI-method* (5.6 ± 0.4 ml/g/min) and the *T1-method* (5.6 ± 0.3 ml/g/min) in our study. As seen in Table 3, our values are also within the range of published values [6,14,17-20], for myocardial perfusion in mice using standard ASL methods. The mean standard deviation of repeated measures by the *SI-method* is 3.6%. This shows that the perfusion quantified using the proposed method is highly reproducible. Interestingly we noted that the variation of *P* within the myocardium was significantly higher for the *T1-method* compared to that of the *SI-method* in the *in vivo* study. A probable cause is the variation in beat to beat time difference in the cardiac cycle resulting errors in the inversion times. This problem is exuberated by the long scan time of the *T1-method*. However, recently introduced post processing techniques could be used to mitigate this error [17]. 

**Table 3:** Published values of myocardial perfusion of healthy mice using CMR methods

**Reference**	**Perfusion (ml/g/min)**
[18] Makowski M. et al., 2010	7.3 ± 0.8 ^*^
[6] Streif JU. et al., 2005	7.0 ± 0.5 ^*^
[19] Nahrendorf M. et al., 2006	6.7 ± 0.3 ^*^
[14] Kober F. et al., 2005	6.0 ± 1.9 ^+^
**Proposed*SI-method***	**5.6 ± 0.5**^**+**^
[17] Vandsburger MH. et al., 2010	5.2 ± 0.8 ^*^
[20] Vandsburger MH. et al., 2007	4.3 ± 0.3 ^*^

^*^ standard error, ^+^ standard deviation.

In general, regions of LGE matched with regions of hypo-perfusion in the ischemia reperfusion study. But in some cases, low perfusion regions extended to the mid-myocardium beyond the boundary of the enhancement (arrows, Figure 3). It is possible that contrast material was not retained in these areas since it was surrounded by normally perfused tissue. It should also be noted that the perfusion maps have a lower resolution than that of the LGE images. Therefore these low perfusion extensions represents some volume averaging.

Although the slice thickness of the inversion pulse was twice that of the excitation pulse we believe it was much less in the actual implementation since the RF pulse shapes were significantly different. The goal was to compensate for miss alignment of the inversion and excitation slice profiles due to motion. However the thicker inversion slice underestimates perfusion. The shorter scan time of the *SI-method* somewhat reduces the errors due to motion thereby allowing for better alignment of the two profiles. This error can be vastly mitigated in human studies where single shot acquisitions can be used. Recently published work by Zun et al. demonstrates the feasibility of such a signal based ASL method with a single shot acquisition in humans [10].

The aim here was to introduce a new theoretical frame work for signal based ASL but we admit that much work remains to be done to improve the imaging technique. The quality of the inversion is problematic for high fields in general where B1 field inhomogeneity is much worse; thus remains a source of error in this application. Transit time effects and coil inflow time effects are associated with improper inversion pulses. These issues were not addressed in this study. We refer the readers to a rigorous analysis of these effects in the context of short TR by Pell et al. [11]. Compared to brain studies physiological noise is another major factor affecting the quality of cardiac perfusion maps. Some tasks for future consideration are: reducing physiological noise, overcoming mis-registration of differently prepared images, achieving a better slice profile for inversion, correcting for varied TI times of different k-space lines. With these improvements in place, the SI-method could potentially be used to generate high resolution pixel-by-pixel perfusion maps in human hearts.

## Conclusion

This study demonstrates that signal intensity based ASL method is a robust alternative to the conventional *T1-method*. The proposed *SI-method* with a segmented acquisition scheme allows faster high resolution perfusion mapping in small animals. Compared to other signal intensity based ASL methods, the two compartmented model used in the present study makes it biologically more accurate.

## Appendix

During repeated inversion pulses at intervals of TR the longitudinal magnetization relaxes as,

(14) $$M_{z}\left(TI\right)=M_{z}\left(0\right)e^{-\frac{TI}{T_{1}}}+M_{0}\left(1-e^{-\frac{TI}{T_{1}}}\right)$$

The 90° excitation pulse saturates the M_z_ and subsequently recovers during the time TR-TI to

(15) $$M_{n}^{-}=M_{0}\left(1-e^{-\frac{TR-TI}{T_{1}}}\right)$$

just before the nth inversion pulse. Therefore just after the nth inversion pulse the magnetization is given by;

(16) $$M_{n}^{+}=-M_{n}^{-}$$

Combining (1) (2) and (3) we get,

(17) $$M_{z}\left(TI\right)=-M_{0}\left(1-e^{-\frac{TR-TI}{T_{1}}}\right)e^{-\frac{TI}{T_{1}}}+M_{0}\left(1-e^{-\frac{TI}{T_{1}}}\right)$$

and

(18) $$M_{Z}\left(TI\right)=M_{0}\left(1-2e^{-\frac{TI}{T1}}+e^{-\frac{TR}{T_{1}}}\right)$$

## Competing interests

The authors declare that they have no competing interests.

## Authors’ contributions

SA: Contributed to the design, mathematical derivations, acquisition and analysis of data, and drafting the manuscript; MS: Performed ischemia reperfusion surgery. JPW: Contributed to the conception and design, supervised acquisition, analysis and interpretation of data, involved in drafting the manuscript. All authors read and approved the final manuscript.

## References

[B1] Bauer WR Hiller KH Roder F Rommel E Ertl G Haase A Magnetization exchange in capillaries by microcirculation affects diffusion-controlled spin-relaxation: a model which describes the effect of perfusion on relaxation enhancement by intravascular contrast agents Magn Reson Med 1996 35 1 43 55 10.1002/mrm.1910350107 8771021

[B2] Northrup BE McCommis KS Zhang H Ray S Woodard PK Gropler RJ Zheng J Resting myocardial perfusion quantification with CMR arterial spin labeling at 1.5 T and 3.0 T J Cardiovasc Magn Reson 2008 10 1 53 10.1186/1532-429X-10-53 19014709 PMC2654036

[B3] Wacker CM Fidler F Dueren C Hirn S Jakob PM Ertl G Haase A Bauer WR Quantitative assessment of myocardial perfusion with a spin-labeling technique: preliminary results in patients with coronary artery disease J Magn Reson Imaging 2003 18 5 555 560 10.1002/jmri.10386 14579398

[B4] Belle V Kahler E Waller C Rommel E Voll S Hiller KH Bauer WR Haase A In vivo quantitative mapping of cardiac perfusion in rats using a noninvasive MR spin-labeling method J Magn Reson Imaging 1998 8 6 1240 1245 10.1002/jmri.1880080610 9848735

[B5] Kober F Iltis I Izquierdo M Desrois M Ibarrola D Cozzone PJ Bernard M High-resolution myocardial perfusion mapping in small animals in vivo by spin-labeling gradient-echo imaging Magn Reson Med 2004 51 1 62 67 10.1002/mrm.10676 14705046

[B6] Streif JU Nahrendorf M Hiller KH Waller C Wiesmann F Rommel E Haase A Bauer WR In vivo assessment of absolute perfusion and intracapillary blood volume in the murine myocardium by spin labeling magnetic resonance imaging Magn Reson Med 2005 53 3 584 592 10.1002/mrm.20327 15723416

[B7] Zhang H Shea SM Park V Li D Woodard PK Gropler RJ Zheng J Accurate myocardial T1 measurements: toward quantification of myocardial blood flow with arterial spin labeling Magn Reson Med 2005 53 5 1135 1142 10.1002/mrm.20461 15844151

[B8] Buxton RB Frank LR Wong EC Siewert B Warach S Edelman RR A general kinetic model for quantitative perfusion imaging with arterial spin labeling Magn Reson Med 1998 40 3 383 396 10.1002/mrm.1910400308 9727941

[B9] Kwong KK Chesler DA Weisskoff RM Donahue KM Davis TL Ostergaard L Campbell TA Rosen BR MR perfusion studies with T1-weighted echo planar imaging Magn Reson Med 1995 34 6 878 887 10.1002/mrm.1910340613 8598815

[B10] Zun Z Wong EC Nayak KS Assessment of myocardial blood flow (MBF) in humans using arterial spin labeling (ASL): feasibility and noise analysis Magn Reson Med 2009 62 4 975 983 10.1002/mrm.22088 19672944

[B11] Pell GS Thomas DL Lythgoe MF Calamante F Howseman AM Gadian DG Ordidge RJ Implementation of quantitative FAIR perfusion imaging with a short repetition time in time-course studies Magn Reson Med 1999 41 4 829 840 10.1002/(SICI)1522-2594(199904)41:4<829::AID-MRM24>3.0.CO;2-U 10332861

[B12] Bauer WR Roder F Hiller KH Han H Frohlich S Rommel E Haase A Ertl G The effect of perfusion on T1 after slice-selective spin inversion in the isolated cardioplegic rat heart: measurement of a lower bound of intracapillary-extravascular water proton exchange rate Magn Reson Med 1997 38 6 917 923 10.1002/mrm.1910380610 9402192

[B13] Bauer WR Schulten K Theory of contrast agents in magnetic resonance imaging: coupling of spin relaxation and transport Magn Reson Med 1992 26 1 16 39 10.1002/mrm.1910260104 1625562

[B14] Kober F Iltis I Cozzone PJ Bernard M Myocardial blood flow mapping in mice using high-resolution spin labeling magnetic resonance imaging: influence of ketamine/xylazine and isoflurane anesthesia Magn Reson Med 2005 53 3 601 606 10.1002/mrm.20373 15723407

[B15] Detre JA Leigh JS Williams DS Koretsky AP Perfusion imaging Magn Reson Med 1992 23 1 37 45 10.1002/mrm.1910230106 1734182

[B16] Richer C Domergue V Gervais M Bruneval P Giudicelli JF Fluospheres for cardiovascular phenotyping genetically modified mice J Cardiovasc Pharmacol 2000 36 3 396 404 10.1097/00005344-200009000-00017 10975599

[B17] Vandsburger MH Janiczek RL Xu Y French BA Meyer CH Kramer CM Epstein FH Improved arterial spin labeling after myocardial infarction in mice using cardiac and respiratory gated look-locker imaging with fuzzy C-means clustering Magn Reson Med 2010 63 3 648 657 10.1002/mrm.22280 20187175 PMC2918386

[B18] Makowski M Jansen C Webb I Chiribiri A Nagel E Botnar R Kozerke S Plein S First-pass contrast-enhanced myocardial perfusion MRI in mice on a 3-T clinical MR scanner Magn Reson Med 2010 64 6 1592 1598 10.1002/mrm.22470 20928891 PMC3179599

[B19] Nahrendorf M Streif JU Hiller KH Hu K Nordbeck P Ritter O Sosnovik D Bauer L Neubauer S Jakob PM Multimodal functional cardiac MRI in creatine kinase-deficient mice reveals subtle abnormalities in myocardial perfusion and mechanics Am J Physiol Heart Circ Physiol 2006 290 6 H2516 H2521 10.1152/ajpheart.01038.2005 16415075

[B20] Vandsburger MH French BA Helm PA Roy RJ Kramer CM Young AA Epstein FH Multi-parameter in vivo cardiac magnetic resonance imaging demonstrates normal perfusion reserve despite severely attenuated beta-adrenergic functional response in neuronal nitric oxide synthase knockout mice Eur Heart J 2007 28 22 2792 2798 10.1093/eurheartj/ehm241 17602202

